# Tick-Borne Encephalitis Risk Increases with Dog Ownership, Frequent Walks, and Gardening: A Case-Control Study in Germany 2018–2020

**DOI:** 10.3390/microorganisms10040690

**Published:** 2022-03-23

**Authors:** Teresa Marie Nygren, Antonia Pilic, Merle Margarete Böhmer, Christiane Wagner-Wiening, Ole Wichmann, Thomas Harder, Wiebke Hellenbrand

**Affiliations:** 1 Immunization Unit, Robert Koch Institute, Seestraße 10, 13353 Berlin, Germany; pilica@rki.de (A.P.); wichmanno@rki.de (O.W.); hardert@rki.de (T.H.); wkhellenbrand@gmail.com (W.H.); 2 Charité-Universitätsmedizin Berlin, 10117 Berlin, Germany; 3 Bavarian Health and Food Safety Authority (LGL), Veterinärstraße 2, 85764 Oberschleißheim, Germany; merle.boehmer@lgl.bayern.de; 4 Faculty of Medicine, Institute of Social Medicine and Health Systems Research, Otto-von-Guericke-University Magdeburg, 39120 Magdeburg, Germany; 5 State Health Office Baden-Wuerttemberg (LGA), Nordbahnhofstraße 135, 70191 Stuttgart, Germany; christiane.wagner-wiening@rps.bwl.de

**Keywords:** tick-borne encephalitis, epidemiology, risk factor, transmission, tick bites, prevention, surveillance, case-control, Germany

## Abstract

In Germany, tick-borne encephalitis (TBE) infections mainly occur in southern regions. Despite recent increases in incidence, TBE vaccination coverage remains low, necessitating additional preventive strategies against TBE. Our case-control study in Southern Germany from 2018 to 2020 mapped knowledge/application of tick-protective strategies and identified TBE risk factors. We calculated odds ratios (OR), with 95% confidence intervals (CI). We interviewed 581 cases and 975 matched controls. Most participants recalled lifetime tick bites, mainly while walking, gardening, or hiking. However, only 45% of cases noticed ticks during exposure time; another 12% reported unpasteurized milk intake. While tick-protection knowledge was satisfactory, application lagged behind. Risk factors included dog ownership (OR = 2.45, 95% CI: 1.85–3.24), walks ≥ 4×/week (OR = 2.11, 95% CI: 1.42–3.12), gardening ≥ 4×/week (OR = 1.83, 95% CI: 1.11–3.02), and garden proximity < 250 m of forests (OR = 2.54, 95% CI: 1.82–3.56). Applying ≥2 tick-protective strategies (OR = 0.52, 95% CI: 0.40–0.68) and keeping lawns mowed (OR = 0.63, 95% CI: 0.43–0.91) were inversely associated with TBE. In 2020 (likely pandemic-related), cases reported significantly more walks than previously, potentially explaining the record high case numbers. Our findings provide guidance on targets for TBE prevention. Persons with gardens near forests, frequent outdoor activities, or dogs could particularly benefit from targeted information, including on vaccination and preventing tick bites.

## 1. Introduction

A marked increase in tick-borne encephalitis (TBE) cases was recently observed in Germany. While the median annual count of routinely notified TBE cases was 276 from 2001 to 2016, this figure almost doubled to 529 from 2017 to 2020 [1]. Of notified cases, 20% experienced severe disease such as meningoencephalitis and 51% had not fully recovered three months after onset [2]. TBE vaccination coverage is low, even in the two federal states where 85% of all TBE cases occur in Germany (22.3% in Bavaria are fully vaccinated; 18.0% in Baden-Wuerttemberg) [1]. To curb the high TBE-related morbidity, it is key to both increase vaccination coverage and to develop additional targeted prevention strategies. 

Effective TBE prevention requires knowledge and awareness of risk factors in at-risk populations, as well as knowledge and application of tick-protective strategies. To our knowledge, risk factors specific to TBE have only been studied in a single case-control study by Stefanoff et al. (2012). In this study, TBE was significantly associated with spending ≥10 weekly hours in forests, living ≤500 m from forests, and socioeconomic factors such as unemployment or occupation as forestry workers [3]. Moreover, TBE seroprevalence was associated with hunting, tourism, fishing, and raw milk intake in Slovenia [4]. While TBE is primarily tick-borne, alimentary transmission is possible via raw (unpasteurized) milk (products) from cows, goats, or sheep [5,6]. In routine surveillance, milk-borne outbreaks are rarely reported, and only 1–2% of German cases mention milk as the likely source of infection [1].

Studies on other tick-borne diseases or on risk factors for tick bites in general might provide further clues for prevention. In Germany, Lyme disease (LD) was associated with gardening, skin contact with greenery, and proximity to forests [7]; and similarly, in the USA, with woodland on property, proximity to forests, and gardening, but no other outdoor activities [8]. Dog and cat ownership were not relevant in the USA and the Netherlands [9,10], yet high proportions of Canadian dog owners had risk factors for ticks, such as frequent outdoor activities [11]. German LD seroprevalence was higher in males and rural residents [12]. 

In a Dutch study, 43% of tick bites occurred in forests and 31% in gardens [13]. A Slovenian study identified walking, mushroom/berry picking, and outdoor sports as tick bite-prone activities [14], but did not mention gardening. Tick-protective strategies are known to be applied by 52–58% of respondents in various settings [11,15,16], but have been hitherto understudied in Germany. 

Further research is necessary, given this partly contradictory body of evidence, the sensitivity of variables to national and ecological contexts, differences between LD and TBE epidemiology, and national differences in behavior and leisure activities. Identifying determinants of tick bites can, moreover, help prevent other tick-borne infections, including LD.

The overall objective of our study was to identify high-risk population groups and risk-related behaviors in Germany, as a basis for developing effective and context-specific measures to improve TBE prevention. In particular, we aimed to (i) assess participants’ awareness of living in official TBE risk areas, where vaccination is recommended and free of charge, (ii) analyze knowledge and application of tick-protective strategies, and (iii) identify further risk factors leading to tick bites, such as outdoor activities.

## 2. Materials and Methods

### 2.1. Study Population and Data Collection

We invited TBE cases notified from 1 January 2018 to 31 December 2020 and living in Bavaria or Baden-Wuerttemberg, and who met the German case definition [17], to participate. Local health authorities established the first contact with cases. Following written consent, data were collected using 30-min standardized telephone interviews (USUMA GmbH, Berlin, Germany). A detailed account of study design and data collection has been published elsewhere [2]. The median time interval between symptom onset and study interviews was 93 days (IQR = 66–146). The main reasons for delay were cases’ hospital stays/rehabilitation and time to respond to the invitation letter.

Population controls were recruited by USUMA GmbH from a representative telephone sample. To be eligible, persons had to speak German and must never have been diagnosed with TBE. Controls were frequency-matched to cases by sex, age (+/−5 years), and 16 geographical regions. Telephone interviews (20 min) were conducted, following verbal consent.

Interviews covered demographics, comorbidities, education, TBE vaccination, and covariates with a potential link to TBE risk. Cases, moreover, provided data on acute clinical symptoms and healthcare use, as reported in [2]. For all cases, data from the routine TBE surveillance system were available, which were compared with information provided by the participants in our study (especially data on tick bites and raw milk intake).

The study was approved by the Ethics Committee of Charité—Universitätsmedizin Berlin, Germany, No. EA2/059/18.

### 2.2. Variable Classification

Frequency of performing eleven risk activities was retrospectively measured on a 5-point Likert scale (never, <1×/week, 1–3×/week, 4–6×/week, daily), referring to the 4-week period prior to symptom onset for cases (exposure time). For controls, questions referred to corresponding 4-week periods, matching the time of year of cases’ respective 4-week periods to adjust for season. Data were transformed into 3-point-categories for analysis. Given the predominance of gardening and taking walks among the 11 measured outdoor activities, the other 9 less frequently reported activities, such as hiking or fishing, were grouped. For individual univariable estimates for all eleven activities, see Supplementary Materials Table S2. 

We identified seven protective strategies against tick bites from expert knowledge and the literature [11]. We included one additional decoy strategy, wearing a hat, to measure the common misconception that ticks drop from trees. Participants were asked whether they considered strategies effective, and if so, whether they regularly applied these in the above-mentioned 4-week period. 

We collected data on additional potential risk factors, including ownership of cats and dogs. Cats living exclusively indoors were not counted. The questionnaire item raw milk (product) intake covered milk (products) from cows, sheep, or goats, and provided examples. 

Vaccination status was defined based on dose number and time interval since last dose (cases: days between last dose and symptom onset, controls: days between last dose and interview) according to manufacturers’ instructions. Detailed vaccination aspects will be reported elsewhere. 

### 2.3. Data Analyses

Data were analyzed in Stata 17^®^. We reported means, medians, and percentages and tested differences with chi-square, Mann–Whitney U, and Kruskal–Wallis tests, as appropriate. *p*-values < 0.05 were considered statistically significant. Where stated, *p*-values < 0.01 were used in multiple comparisons. 

For risk factor analysis, we excluded participants not living or spending time in TBE endemic areas (*n* = 5 cases, 11 controls) and fully, on-time vaccinated participants (*n* = 15 cases, 234 controls). We explored the underlying causal structure with directed acyclic graphs (DAGs) in Dagitty [18], to identify the minimal adjustment sets required to estimate the total causal effect for each exposure of interest on TBE risk (Supplementary Materials Figure S1. We report odds ratios (OR) with 95% confidence intervals (CI), each adjusted for the exposure-specific minimal adjustment set (Supplementary Materials Table S1). For univariable estimates, see Supplementary Materials Table S2. A sub-analysis restricted to participants with garden access further explored garden properties. 

We also compared cases’ frequency of performing risk behaviors, as identified from a risk factor analysis, between study years 2018–2019 and 2020, the first year of the coronavirus disease 2019 (COVID-19) pandemic. In also drawing on data from [2], we lastly tested two hypotheses for why TBE incidence is higher in males than females. 

## 3. Results

### 3.1. Study Population

Of 1220 eligible TBE cases, 581 (48%) participated, without evidence of selection bias (see also [2]). Hospitalization was reported for 90% of cases, with a 12.3 day mean duration of stay. The 975 participating controls (1.7 per case) resembled cases regarding the matching factors age, sex, and region, but differed in vaccination status and potential risk factors (Table 1). Almost all participants lived in risk areas (Table 1), yet many were not aware of this (controls: 35.6% unaware, cases: 40.5% unaware before contracting TBE, *p* = 0.06). TBE vaccination coverage (≥1 dose) was significantly lower in controls unaware of risk areas than in those aware (42.6% vs. 69.5%, *p*_Chi2_ < 0.001). This difference was less pronounced among cases (11.1% vs. 15.7%, *p*_Chi2_ = 0.228).

### 3.2. Tick Bites, Activities Leading to Tick Bites, and Raw Milk Intake

Cases reported more tick bites than controls (Table 1). Most participants with at least one lifetime tick bite (1081 of 1130 persons, 95.7%) recalled the activities during which bites occurred, most commonly taking walks, gardening, and hiking. Activities were similar in cases and controls (Table 2).

Among cases, 250 (44.8%) noticed at least one tick bite during the specified exposure time. Of these, 39 cases additionally reported raw milk intake during the exposure time. Sixty-six cases (11.8% overall) reported raw milk intake but no ticks, and 242 cases (43.4%) had no known source of infection. There was no statistically significant difference in recalled tick bites by sex (43.5% men vs. 47.1% women, *p* = 0.289). Bites were more frequently reported for children than adults, (58.1% vs. 43.2%, *p* = 0.081). Most cases with bites (61.0%) reported a single bite, 33.3% two to five bites, and 5.7% up to 30 bites during exposure time. Among 151 cases reporting a plausible tick bite date (60.4% of 250 cases reporting exposure period-bites), the median duration between (last) bite and symptom onset was 7 days (IQR = 2–16 days). 

In routine surveillance data, reporting of tick bites was congruent for 223 of the 250 study cases with tick bites (89.2%); no tick bite was reported for 6 cases (2.4%) and 21 had missing data (8.4%). Among the 307 cases reporting no tick bites during interviews, surveillance data were congruent in only 108 cases (35.5%), with erroneously reported tick bites in 109 (35.5%) and missing data in 90 cases (29.3%). 

### 3.3. Tick Removal Practices

Most cases with tick bites during the exposure time removed ticks within one hour of noticing them (86.8%), either themselves (53.6%) or helped by another person (33.2%). This was comparable to cases with tick bites outside of the exposure time and controls regarding earlier tick bites (79.0% and 80.9%, respectively, *p* = 0.225). Other persons removing ticks were predominantly partners (48.5%) or medical personnel (28.9%). Parents usually removed ticks in the case of children (86.6%). The most common reasons given by the 165 participants reporting delayed removal were not noticing the tick earlier (47.3%), waiting for someone else (30.9%), and not having a suitable tool to hand (11.5%). Less common reasons included uncertainty regarding removal techniques (4.2%), fear of failing (3.6%), fear of the head remaining stuck (1.8%), and disgust (*n* = 0). 

The size of ticks removed within one hour (*n* = 903) was, according to cases, smaller than a pepper grain (67.2%), the size of a pepper grain (29.2%), or pea-sized (3.5%). Ticks removed after >1 h (*n* = 151), were significantly larger: 56.3%, 33.8%, 9.9%, respectively (*p*_Chi2_ = 0.001).

### 3.4. Risk Factors for TBE

**Figure 1:**
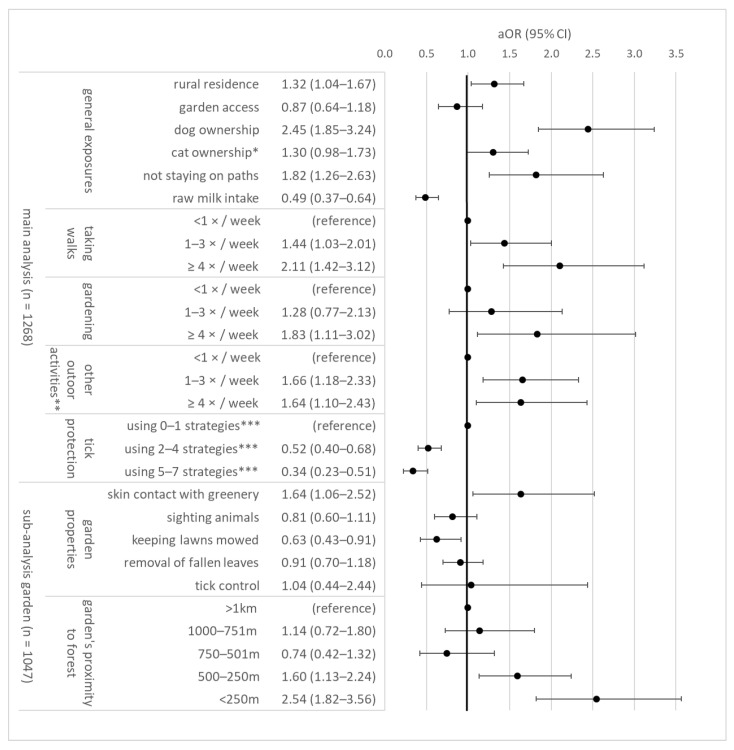
Risk and protective factors for TBE, *n* = 1268 study participants (538 TBE cases, 730 controls) from Southern Germany. The sub-analysis only included persons with garden access (*n* = 1047). Estimates represent the total causal effect for each covariate, adjusted for matching factors and covariate-specific minimal adjustment sets of variables (Supplementary Materials Table S1 and Figure S1). For univariable estimates, see Supplementary Materials Table S2. aOR = adjusted odds ratio; CI = confidence interval. * only outdoor cats. ** activities include (in descending frequency): biking, hiking, running/Nordic Walking, other outdoor sport, forestry/logging, bird watching, fishing, hunting, bee keeping. *** regularly applying tick-protective strategies (e.g., tick repellant) during exposure time (cases) or reference time (controls).

Dog ownership, taking walks, gardening, not staying on paths, other outdoor activities, and rural residence in settlements with <5000 inhabitants were significantly associated with TBE (Figure 1). Applying tick-protective strategies and raw milk intake were negatively associated with TBE risk. Dose-response effects were often observed, e.g., walks >4 times a week was more strongly associated with risk than 1–3 times a week. Hunting was associated in the univariable analysis (Supplementary Materials Table S2).

While garden access was not associated with TBE risk, a sub-analysis revealed an association of TBE with skin contact with greenery and a garden’s proximity to forests. Risk was reduced when lawns were mowed regularly, while regular removal of fallen leaves, tick control, and sighting animals were not associated with TBE risk. Concerning specific animal species, cases more frequently sighted mice (7.5% vs. 1.3%, *p*_Chi2_ < 0.001), moles (12.7% vs. 6.5%, *p*_Chi2_ = 0.001), and deer (30.4% vs. 22.8%, *p*_Chi2_ = 0.01) than controls, but less frequently other forest animals (5.2% vs. 10.7%, *p*_Chi2_ = 0.004). For details on other animal species, see Supplementary Materials Table S3.

A sex-stratified sensitivity analysis of risk factors produced similar results (data not shown). When stratifying the dataset as aged above (*n* = 417) or below 60 years (*n* = 851), results remained robust, except other outdoor activities ≥4×/week and rural residence, which were associated with TBE in persons older than 60 years (outdoor OR = 2.96, 95% CI: 1.44–6.07; rural OR = 2.02, 95% CI: 1.33–3.07), but not in younger persons (outdoor OR = 1.21, 95% CI: 0.74–1.97; rural OR = 1.14, 95% CI: 0.69–1.88).

In a further sensitivity analysis, the arrow in the DAG (Supplementary Materials Figure S1) pointed from outdoor activities to dog ownership instead of vice-versa; thus, requiring additional covariate adjustment for walks and other outdoor activities. The resulting OR for dog ownership was slightly lower at 1.86 (95% CI: 1.37–2.52).

Among dog owners, we compared potentially infection-relevant factors between cases (*n* = 162) and controls (*n* = 112). Groups were comparable regarding regularly walking dogs (cases 90.1%, controls 88.4%, *p*_Chi2_ = 0.65), applying tick-protective measures on dogs (cases 85.8%, controls 82.1%, *p*_Chi2_ = 0.21), or noticing ticks on dogs within 4 weeks (cases 68.5%, controls 75.9%, *p*_Chi2_ = 0.29). Proportions noticing ticks on cats were also similar among cat owners (cases 54.8%, controls 61.4%, *p*_Chi2_ = 0.40). Notably, dog owners tended to report more tick bites than non-dog owners (*p*_Chi2_ = 0.068, e.g., 33.9% of dog-owning controls vs. 24.6% controls without dogs had ticks in 12 months). 

Further exploration of raw milk intake (see Table 1), revealed that product type did not differ between cases and controls: of 407 persons, 21.9% consumed only raw milk, 51.8% only raw dairy products, and 26.0% both. Raw milk was consumed at all ages. 

Occupations with potential tick exposure were not associated with TBE (see Supplementary Materials Table S2), but reported by 77 cases (22.9%) and 112 controls (18.5%, *p* = 0.096) in employment. TBE vaccination was similar to that of persons without occupational exposure among both groups. Common professions were handyman (25.9%), teacher/educator (20.1%), agriculture worker (19.6%), gardener (9.5%), and forestry worker (5.8%). 

### 3.5. TBE Risk Behavior during the COVID-19 Pandemic

**Figure 2:**
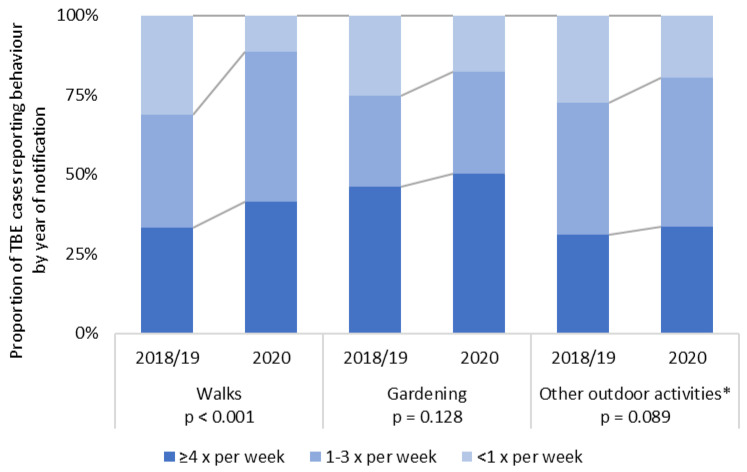
Proportion of TBE cases (*n* = 558) reporting outdoor behaviors during the 4-week exposure period. The study period was split into years before and after the beginning of the COVID-19 pandemic. Chi-square test results are reported within the graph. * activities include (in descending frequency): biking, hiking, running/Nordic Walking, other outdoor sport, forestry/logging, bird watching, fishing, hunting, bee keeping.

Cases from 2020 reported significantly more frequent outdoor walks than cases from 2018–2019 (Figure 2). There were non-significant tendencies towards more frequent gardening and other outdoor activities in 2020. Other relevant factors from the risk factor analysis did not differ significantly between years (Supplementary Materials Table S4). Hospitalization and symptoms reported in routine data were also similar between years.

### 3.6. Tick-Protective Strategies

**Figure 3:**
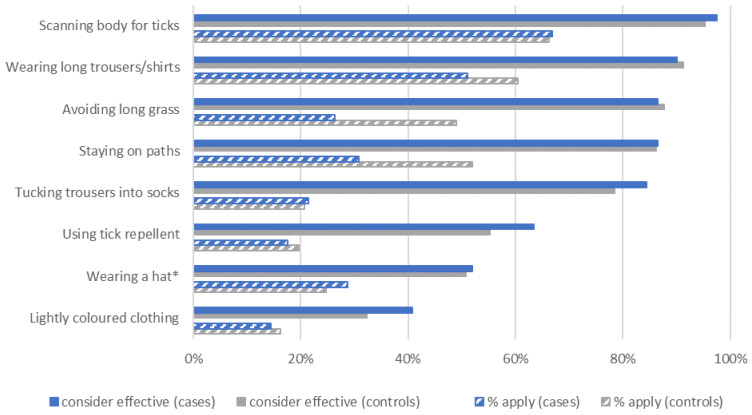
Knowledge and application of tick-protective strategies in TBE cases (*n* = 558) and controls (*n* = 975). Data on applying strategies was only collected among participants considering a strategy effective. * deliberate decoy question, as ticks do not drop from trees. The covariate ‘tick protection’ used in risk factor analysis only considers the 7 relevant strategies.

Knowledge of tick-protective strategies was similar in cases and controls (Figure 3). However, markedly more controls reported applying strategies, particularly avoiding long grass and staying on paths (difference to cases: >20%, *p*_Chi2_ < 0.001), as well as wearing long trousers/shirts (>9% difference, *p*_Chi2_ < 0.001).

### 3.7. Exploring Why TBE Incidence Is Higher in Males Than Females

We tested two hypotheses of why TBE incidence is 1.7 times higher in males than in females [19]. Hypothesis 1 is that males seek healthcare earlier, with milder symptoms. This would lead to higher male case numbers, with overall milder disease. Severity, as assessed in the preceding publication within this project [2], was however similar between sexes (males: 19.6% mild, females: 20.2% mild, *p*_Chi2_ = 0.98). Likewise, the proportion hospitalized (males: 91.0%, females: 87.3%, *p* = 0.16) and mean delay between symptom onset and hospital admission (males: 12.6 days (SD = 11.4), females 13.9 days (SD = 10.6), *p* = 0.06) were similar. 

Hypothesis 2 is that men are more prone to risk behaviors, resulting in more infections. However, women more frequently reported the risk factors with the largest effect sizes than men: dog ownership (33.3% women vs. 27.9% men, *p*_Chi2_ = 0.18), walks > 4×/week (41.7% vs. 33.3%), gardening > 4×/week (52.5% vs. 44.9%, *p*_Chi2_ = 0.31), and not staying on paths (25.5% vs. 22.9%, *p*_Chi2_ = 0.60). Notably, women reported applying more protective measures (≥2 measures: 73.0% vs. 61.0%, *p*_Chi2_ = 0.004).

## 4. Discussion

By comprehensively studying 581 TBE cases and 975 population controls, this study revealed gaps in awareness and implementation of preventive measures, as well as novel behavioral and environmental risk factors for TBE. These could be targeted in future prevention efforts. A main insight is that almost 40% of participants were unaware of living in official TBE risk areas; and among these, vaccination coverage was particularly low. Even though TBE vaccination is officially recommended and free of charge in these areas, coverage is ~20% [1]. Improving awareness could lead to improved vaccination coverage.

Only 45% of cases reported tick bites during the specified exposure time and a further 12% reported raw milk intake. Most remaining cases (43%) likely had unnoticed tick bites. Tick bites were reliably reported in routine surveillance for cases who also reported bites during study interviews, but overreported in a third of cases who did not report bites in interviews. According to German surveillance data, 61–66% of cases reported tick bites [1,20]. This overreporting may stem from imprecisions in routine data collection, e.g., if also counting tick bites outside the exposure time. Previously reported proportions range from 58–82% [14,21]. In resource-limited settings, a prioritization of TBE diagnostics only for patients with tick bites has been suggested [22]. Given our findings of only 45% tick bite recall, such prioritization would lead to marked under-recognition of TBE. Instead, we suggest that TBE should be diagnostically considered in any person with TBE-compatible symptoms, regardless of tick bite recall, particularly in risk areas.

Tick bites mainly occurred during walks, gardening, hiking, and forestry/logging. These findings are congruent with previous reports [13], but highlight that, contrasting [14], private gardens are key sources of tick bites in Germany. 

Tick removal was performed largely as recommended and similar to previous publications reporting swift tick removal in 89–97% [14,16,23]: most cases (87%) removed ticks within one hour. Swift removal reduces LD risk [24]. However, late tick removal was not associated with higher TBE severity [2], and it remains uncertain if blood meal duration also impacts TBE transmission and pathology, e.g., by decreasing viral load [25]. Tick-related disgust and lack of tick removal confidence did not have an effect on tick removal time, contrasting with earlier reports [26]. Notably, 34% of the reported promptly removed ticks in our study were pepper grain- or pea-sized, i.e., they had likely already been attached for hours (or even days) before detection. Failure to notice ticks as the main reason for delayed removal underlines that scanning the body for ticks after potential exposure is crucial. 

The activities during which tick bites occur (Table 2) are informative for public health awareness campaigns. Applying tick-protective strategies during these activities could improve prevention, not only of TBE, but of other tick-borne infections such as LD or tularemia. Our further multivariate, adjusted analyses more robustly identified TBE-specific risk factors: dog ownership, taking walks, gardening, not staying on paths, other outdoor activities, rural residence, garden’s proximity to forest, and skin contact with garden greenery. Several associations were frequency-dependent, strengthening confidence in their robustness. 

Our results agree with a Polish case-control study [3], which also identified proximity to forest and spending time outdoors as TBE risk factors. However, gardening was not associated in their study. Socioeconomic parameters were central in the Polish setting, yet in our study occupational exposure was not associated, and education only weakly associated, with TBE (Supplementary Materials Table S2). Notably, the commonly reported professions here, handymen and teachers, appear to be off the radar and should, in addition to forestry workers, be targeted in future TBE prevention. 

Comparing our results to previous studies, rural residence was not only a risk factor in our analysis, but also associated with LD seroprevalence in Germany [12]. Rural populations are less informed and apply less tick prevention strategies according to a US study [27], which could explain these findings. Observing that dog ownership doubles the odds for TBE is to our knowledge a novel finding. Results contrast previous studies on pets and LD risk [9,10]. Dog owners tended to report more ticks, suggesting a potential mechanism through which dogs may increase risk. Given the high prevalence of dog ownership, future research should re-investigate this issue.

Keeping lawns mowed, a strategy adopted by 65% of the Canadian population to mitigate LD risk [11], was protective in our analysis. Targeted information campaigns to German garden owners may increase this strategy’s implementation. 

Cautious interpretation is essential regarding the observed protective effect of tick-protection strategies, as differential respondent bias may be present. Cases may have underestimated their degree of applied protection—after all, they contracted TBE infection—while controls may have overreported, due to social desirability bias. As it is, results imply that regularly applying 2–4 strategies may reduce risk to half, yet the unbiased effect may be smaller. On the other hand, selecting the most effective strategies rather than just counting the number of applied strategies may yield stronger effects. For instance, the effectiveness of long clothing is undisputed [11,28], while lightly colored clothing may in fact attract more ticks [29], rendering this strategy counterproductive. Experimentally testing strategies’ comparative effectiveness would be valuable, to develop evidence-based recommendations on which strategies to use. 

Raw milk intake by 31% of controls and 19% of cases was unexpectedly common, given the known infection risk [5,6,30]. As some cases additionally reported tick bites, milk was considered the possible source of infection in 66 cases (12%). Some of these 66 likely had unnoticed tick bites. This figure exceeds the routinely reported 1–2% [1] and may be attributable to the precise wording in our questionnaire. Milk-borne TBE transmissions seem underreported in German routine surveillance. We suggest improving the wording in routine data collection and increasing follow-up investigations on milk-related transmissions. A hypothetical explanation for the observed negative association of raw milk with TBE may be that persons growing up regularly consuming raw milk in TBE-endemic areas might experience mild or asymptomatic, unnoticed TBE infections at early ages; thus acquiring long-lasting immunity. If consumption habits continued into adulthood, this might explain the observed higher proportion of controls with raw milk intake. Future studies could test this theory by correlating TBE seroprevalence with lifetime raw milk intake. Alternative explanations for the observed case-control difference in reported milk intake are conceivable, e.g., differential recall bias if more cases than controls did not recall raw milk intake, due to more salient memories of tick bites during exposure time.

Our data suggests that the record high of 712 notified TBE cases in Germany in 2020 may be attributable to significantly more frequent outdoor walks—permitted under COVID-19-related restrictions—compared to 2018–2019. Other studies on changes in physical activity during lockdown reported mixed results [31]. Other factors contributing to high infection rates may have included high tick abundance. COVID-19-related limitations in diagnostic or surveillance capacity, as in Poland [32], do not seem to have produced a diagnostic bias towards severe cases in Germany, as hospitalization rate and symptoms remained unchanged. 

Participants’ knowledge of tick-protective measures was mainly satisfactory. Protective measures’ perceived efficacy increased their use [33], yet their reported application markedly lagged behind knowledge. Our figures lie within, or above, the published ranges; e.g., the 66% application of tick inspection slightly exceeds the published 52–58% [11,15,16]. A bias towards socially desirable responses is likely; hence, true numbers may be lower. As the knowledge on strategy effectiveness is already high, public health efforts should focus on motivating the population to apply such strategies. 

The data did not support the two hypotheses for why TBE incidence is higher in males. Severity was equal between sexes, speaking against differing health seeking behavior. Nor were key risk factors more common among men than women. Given our data, we could, however, not measure unnoticed tick bites, which may be more common among men, as also indicated by the higher use of tick protection among women. This aspect and other possible reasons for the difference in incidence, e.g., sex-specific disease manifestations due to hormonal differences, should be explored further. 

Limitations, first, include that almost all data in this analysis were self-reported. Differential reporting biases may apply, for instance concerning exposure-time behaviors, such as tick-protective behaviors. Other covariates, including dog ownership or rural residence, were not prone to this bias. Second, the sampling method did not allow estimating a response rate for controls. Participating controls may be more aware of TBE risk than the general population, which would increase differences to cases. TBE vaccination, indicative of TBE awareness, was slightly higher among controls (24.1%) than in the general population (18.0–22.3% [1]). As this difference is only moderate, and case-control comparability was otherwise good (Table 1), the possible bias is likely small. Third, the risk factor analysis is dependent on the assumed underlying causal structure and covariate adjustment sets. Assumptions were diligently discussed with experts, yet perfect adjustment can never be achieved. We made assumptions as transparent as possible, through Supplementary Materials Figure S1, Tables S1 and S2, and tested alternatives in sensitivity analyses. Thus, the here reported results are, to our knowledge, the optimally adjusted estimates. 

The main strengths of our study include the high level of detail, covering manifold important aspects concerning TBE risk and prevention. Thus, we not only identified risk conferred by gardening, but were able to report detailed garden properties, to better understand which aspects may underlie TBE risk from gardening. A second strength is our large sample size, which allowed stratification by age and sex for sensitivity analyses and improved confidence in our findings’ robustness. Third, our comprehensive approach produced ready-to-use insights, which can help design improved preventive strategies, targeting specific areas where current practices are suboptimal and TBE risk is highest.

## 5. Conclusions

We examined a large sample of 581 TBE cases, revealing that only 45% of cases noticed tick bites, that transmission via raw milk may be more common than assumed, and that tick bites not only occur near forests, but commonly in private gardens. Moreover, only about 60% of participants living in TBE risk areas were aware of this. While most subjects were well informed about tick-protective strategies and removed ticks quickly, substantial potential remains for increasing the application of protective strategies. Moreover, we identified dog ownership, taking walks, gardening, not staying on paths, other outdoor activities, and garden’s proximity to forests (<500 m) as TBE risk factors. An increase in taking walks due to COVID restrictions may explain the record case numbers in 2020. Protective effects emerged for applying tick-protective strategies and keeping lawns mowed. Taken together, our findings can be used to design targeted intervention strategies aimed at reducing TBE transmission risk and, thus, improving prevention. Some findings, including a potentially protective effect of raw milk, call for further research.

## Figures and Tables

**Table 1:** Characteristics of study participants: demographics, TBE vaccination status, and selected potential risk factors for TBE.

	Cases	Controls	*p*-Value
	*n* = 581 ^a^	*n* = 975	
	n (%)	n (%)	
**Demographics**			
Age in years (mean and standard deviation)	48.6 (20)	49.9 (19)	0.337
Age group < 18 years	66 (11%)	83 (9%)	0.176
Age group 18–65 years	406 (70%)	698 (72%)	
Age group > 65 years	109 (19%)	194 (20%)	
Male	368 (63%)	608 (62%)	0.699
≥1 comorbidity (self-reported)	118 (21%)	236 (24%)	0.172
Home in TBE risk area	565 (97%)	945 (97%)	0.716
Highest level of completed secondary education *			
Abitur (12–13 years)	162 (29%)	307 (31%)	0.049
Fachabitur (12–13 years)	55 (10%)	81 (8%)	
Realschulabschluss (10 years)	142 (25%)	300 (31%)	
Hauptschulabschluss (9 years)	133 (24%)	194 (20%)	
Still in school/none/missing	66 (12%)	93 (10%)	
**TBE vaccination**			
Unvaccinated (for cases: before onset)	503 (87%)	397 (41%)	<0.001
Partial (<3 doses/time interval too long/details missing)	61 (10%)	343 (35%)	
Complete (≥3 doses) and on-time	17 (3%)	235 (24%)	
**Selected potential TBE risk factors**			
Rural residence (<5000 inhabitants)	268 (48%)	413 (42%)	0.095
Tick bite: never	103 (18%)	300 (31%)	<0.001
Tick bite: last bite > 1 year ago	87 (16%)	407 (42%)	
Tick bite: 1–2 bites in last year	198 (35%)	181 (19%)	
Tick bite: ≥3 bites in last year	170 (30%)	87 (9%)	
Dog ownership	167 (30%)	147 (15%)	<0.001
Cat ownership (only outdoor cats)	150 (27%)	215 (22%)	0.033
Occupational exposure ^b^	77 (23%)	112 (18%)	0.096
Using ≥ 2 tick protective strategies	365 (65%)	774 (79%)	<0.001
Garden’s distance to forest <500 m ^c^	266 (58%)	327 (40%)	<0.001
Gardening ≥ 4×/week ^d^	266 (48%)	400 (41%)	0.037
Taking walks ≥ 4×/week ^d^	203 (36%)	176 (18%)	<0.001
Other outdoor activity ≥ 4×/week ^d^	179 (32%)	253 (26%)	0.01
Not staying on paths when walking, e.g., through meadows or underbrush ^d^	133 (24%)	100 (10%)	<0.001
Raw milk (-product) intake ^e^	105 (19%)	302 (31%)	<0.001

^a^ 558 of 581 cases and all controls had interview data, solely used as a denominator for interview-derived variables (education and all ‘Selected potential TBE risk factors’). ^b^ Proportions among employed persons (335 cases, 607 controls). ^c^ Proportions among persons with garden access (462 cases, 825 controls). ^d^ Cases: within 4 weeks before onset, controls: during reference time (see Methods). Further analysis used 3 levels for frequency-graded covariates: <1×/week, 1–3×/week, ≥4×/week. ^e^ Cases: intake within 2 weeks before symptom onset, controls: during reference time (see Methods). * English translations: Abitur = general qualification for university entrance; Fachabitur = subject-related entrance qualification; Realschulabschluss = intermediate school-leaving certificate; Hauptschulabschluss = completion of compulsory basic secondary schooling.

**Table 2:** Self-reported activities during which tick bites occurred for TBE cases and population controls recalling at least one lifetime tick bite (*n* = 434 cases, 647 controls) from Southern Germany. Multiple answers were possible. The significance level is *p* < 0.01 (multiple comparisons).

Activity	Cases		Controls		*p*-Value For	Total	
	*n* = 434		*n* = 647		Difference	*n* = 1081	
	*n*	%	*n*	%	*p*	*n*	%
Taking a walk	139	32.0%	198	30.6%	0.62	337	31.2%
Gardening	109	25.1%	170	26.3%	0.67	279	25.8%
Hiking	86	19.8%	133	20.6%	0.77	219	20.3%
Forestry/logging	67	15.4%	85	13.1%	0.25	152	14.1%
Mushroom picking	18	4.1%	41	6.3%	0.12	59	5.5%
(Mountain-) biking	27	6.2%	20	3.1%	0.01	47	4.3%
Spending time in forest	20	4.6%	14	2.2%	0.02	34	3.1%
Running/Nordic Walking	11	2.5%	23	3.6%	0.35	34	3.1%
Camping	12	2.8%	12	1.9%	0.32	24	2.2%
Other outdoor sport	8	1.8%	12	1.9%	0.99	20	1.9%
Other outdoor activity	10	2.3%	9	1.4%	0.26	19	1.8%
Fishing/being near water	2	0.5%	15	2.3%	0.02	17	1.6%
Hunting	6	1.4%	6	0.9%	0.48	12	1.1%
Berry picking	5	1.2%	7	1.1%	0.91	12	1.1%
Probably from pet	3	0.7%	7	1.1%	0.04	10	0.9%
Other activity	6	1.4%	2	0.3%	0.29	8	0.7%

## Data Availability

The data presented in this study are available on reasonable request from the corresponding author. The data are not publicly available, due to ethical and data privacy protection obligations.

## Supplementary Materials

The following supporting information can be downloaded at: https://www.mdpi.com/article/10.3390/microorganisms10040690/s1, Table S1: Risk and protective factors for TBE: Additional data on frequency of covariates and covariate-specific minimal adjustment sets; Figure S1: Directed acyclic graph (DAG) of the causal structure underlying the mechanisms connecting potential exposures to the outcome TBE; Table S2: Results from univariable analysis on factors potentially associated with TBE risk; Table S3: Animal species sighted in gardens, as reported by TBE cases and controls; Table S4: Proportion of TBE cases (*n* = 558) reporting selected factors associated with TBE in 2020 compared to 2018/2019.
