# Correction: Nygren et al. Tick-Borne Encephalitis Risk Increases with Dog Ownership, Frequent Walks, and Gardening: A Case-Control Study in Germany 2018–2020. *Microorganisms* 2022, *10*, 690

**DOI:** 10.3390/microorganisms12050958

**Published:** 2024-05-10

**Authors:** Teresa Marie Nygren, Antonia Pilic, Merle Margarete Böhmer, Christiane Wagner-Wiening, Ole Wichmann, Thomas Harder, Wiebke Hellenbrand

**Affiliations:** 1 Immunization Unit, Robert Koch Institute, Seestraße 10, 13353 Berlin, Germany; 2 Charité-Universitätsmedizin Berlin, 10117 Berlin, Germany; 3 Bavarian Health and Food Safety Authority (LGL), Veterinärstraße 2, 85764 Oberschleißheim, Germany; 4 Faculty of Medicine, Institute of Social Medicine and Health Systems Research, Otto-von-Guericke-University Magdeburg, 39120 Magdeburg, Germany; 5 State Health Office Baden-Wuerttemberg (LGA), Nordbahnhofstraße 135, 70191 Stuttgart, Germany

In the original publication [1], there was a mistake in omitting one affiliation for the first author, Teresa M. Nygren. Another affiliation, “Charité-Universitätsmedizin Berlin, 10117 Berlin, Germany”, needs to be added for this author.

The correct authorship of this paper should be:

Teresa Marie Nygren ^1,2,^*, Antonia Pilic ^1^, Merle Margarete Böhmer ^3,4^, Christiane Wagner-Wiening ^5^, Ole Wichmann ^1^, Thomas Harder ^1^ and Wiebke Hellenbrand ^1^

^1^  Immunization Unit, Robert Koch Institute, Seestraße 10, 13353 Berlin, Germany; pilica@rki.de (A.P.); wichmanno@rki.de (O.W.); hardert@rki.de (T.H.); wkhellenbrand@gmail.com (W.H.)
^2^  Charité-Universitätsmedizin Berlin, 10117 Berlin, Germany
^3^  Bavarian Health and Food Safety Authority (LGL), Veterinärstraße 2, 85764 Oberschleißheim, Germany; merle.boehmer@lgl.bayern.de
^4^  Faculty of Medicine, Institute of Social Medicine and Health Systems Research, Otto-von-Guericke-University Magdeburg, 39120 Magdeburg, Germany
^5^  State Health Office Baden-Wuerttemberg (LGA), Nordbahnhofstraße 135, 70191 Stuttgart, Germany; christiane.wagner-wiening@rps.bwl.de

*  Correspondence: nygrent@rki.de; Tel.: +49-30-18754-3577

The authors state that the scientific conclusions are unaffected. This correction was approved by the Academic Editor. The original publication has also been updated.
