# Supplementary material for: Tick-Borne Encephalitis Risk Increases with Dog Ownership, Frequent Walks, and Gardening: A Case-Control Study in Germany 2018–2020
Source: Microorganisms. 2022 Mar 23;10(4):690. doi: 10.3390/microorganisms10040690 (PMC9025759; doi:10.3390/microorganisms10040690)
Supplement: Supplementary file 1 [file microorganisms-10-00690-s001.zip › microorganisms-1635027-supplementary.pdf]

microorganisms

MDPI

Supplementary materials

Table S1. Risk and protective factors for TBE: Additional data on frequency of covariates and covariate-specific minimal adjustment sets, as determined with a directed acyclic graph (Supplement Figure S1), n = 1,268 (538 TBE cases, 730 controls).

Exposure of interest main analysis (n = 1,268)		Participants with outcome	Cases with outcome	Minimal adjustment sets in addition to matching factors age, region, sex
general exposures	rural residence	564	260	none
	garden access	1050	443	rural residence
	dog ownership	274	162	cat, rural residence
	cat ownership	304	146	dog, rural residence
	not staying on paths	203	129	taking walks, other outdoor activities
	raw milk intake	329	101	rural residence
gardening	< 1× / week (reference)	303	122	garden access, season
	1-3 × / week	425	160	
	≥ 4× / week	539	255	
taking walks	< 1× / week (reference)	403	124	partial TBE vaccination, cat, dog, garden access, general tick bites, rural residence, season
	1-3 × / week	541	220	
	≥ 4× / week	324	194	
other outdoor activities*	< 1× / week (reference)	378	131	partial TBE vaccination, cat, dog, garden access, general tick bites, rural residence, season
	1-3× / week	537	236	
	≥ 4× / week	353	171	
tick protection	using 0-1 strategies**	344	188	season
	using 2-4 strategies**	767	303	
	using 5-7 strategies**	157	47	
**Exposure of interest sub-analysis on garden (n = 1,047)**		**Participants with outcome**	**Cases with outcome**	**Minimal adjustment sets in addition to matching factors age, region, sex**
garden properties	skin contact with greenery	939	406	garden access
	sighting animals	789	332	garden access, garden's proximity to forest
	keeping lawns mowed	894	366	garden access, skin contact with greenery
	regular removal of fallen leaves	624	262	garden access, skin contact with greenery
	tick control	23	10	garden access, skin contact with greenery
garden's proximity to forest	> 1km	366	124	garden access, rural residence
	1000-751 m	108	41	
	750-501 m	71	21	
	500-250 m	244	110	
	< 250 m	256	146	

*activities include (in descending frequency): biking, hiking, running/Nordic Walking, other outdoor sport, forestry/logging, bird watching, fishing, hunting, bee keeping.

**regularly applying tick-protective strategies (e.g. tick repellents) during exposure time (cases) or reference time (controls).

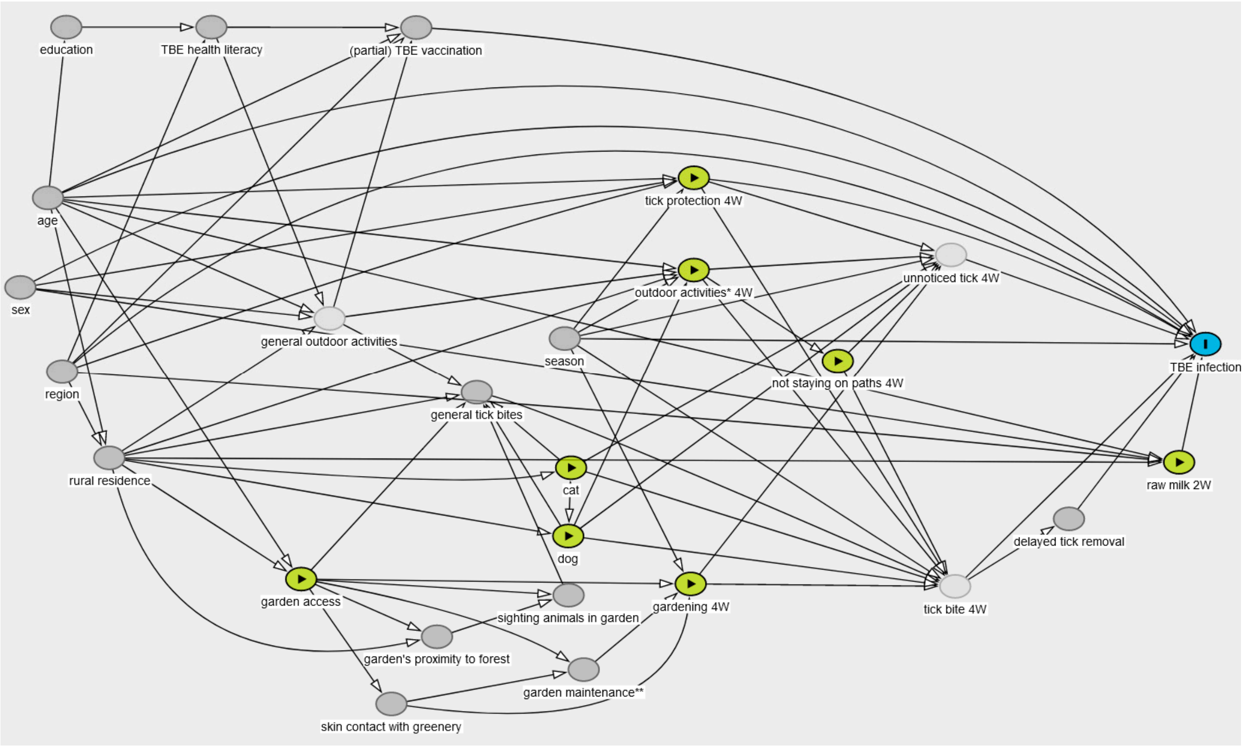

Figure S1. Directed acyclic graph (DAG) of the causal structure underlying the mechanisms connecting potential exposures to the outcome TBE, as determined by subject-specific knowledge. Created with Dagitty [18].

4W = refers to 4-week period of exposure time (cases) or reference period (controls). 2W = refers to 2-week period. Light grey nodes = unobserved parameters. Blue node = outcome. Green nodes = exposures of interest. One exposure was selected at a time to identify the minimal sufficient adjustment set of covariates to estimate the adjusted total causal effect of that exposure on the outcome (for adjustment sets see Supplement 1). The sub-analysis on garden properties considered skin contact with greenery, sighting animals in garden, garden maintenance and garden's proximity to forest as exposures of interest.

* includes both taking walks and other outdoor activities

** includes keeping lawns mowed, regular removal of fallen leaves, tick control.

Table S2. Results from univariable analysis on factors potentially associated with TBE risk. *n* =1,268 (538 TBE cases, 730 controls) from Southern Germany. Results for the sub-analysis on garden properties (covariates in 'garden properties' and 'garden's proximity to forest') only included persons with garden access, *n*=1,047.

Input parameters		OR	Lower 95% CI	Upper 95% CI	p-value
	rural residence	1.31	1.04	1.64	0.020
	garden access	0.94	0.70	1.27	0.706
	dog ownership	2.38	1.81	3.12	< 0.001
	cat ownership	1.35	1.04	1.75	0.024
	occupational exposure	1.22	0.86	1.73	0.256
	not staying on paths	2.78	2.04	3.80	< 0.001
	raw milk intake	0.51	0.39	0.67	< 0.001
Education****	Abitur (12–13 years)	1.00			
	Fachabitur (12–13 years)	1.33	0.87	2.03	0.190
	Realschulabschluss (10 years)	0.89	0.66	1.19	0.414
	Hauptschulabschluss (9 years)	1.32	0.97	1.80	0.080
	still in school/none/missing	1.76	1.16	2.66	0.008
Gardening	< 1× / week	1.00			

	1–3× / week	0.90	0.66	1.21	0.475
	≥ 4× / week	1.33	1.00	1.77	0.049
Taking walks	< 1× / week	1.00			
	1–3× / week	1.54	1.17	2.02	0.002
	≥ 4× / week	3.36	2.47	4.56	< 0.001
Other outdoor activities*	< 1× / week	1.00			
	1–3× / week	1.48	1.13	1.94	0.005
	≥ 4× / week	1.77	1.32	2.39	< 0.001
Individual, non-grouped outdoor activities**	Gardening	1.42	1.13	1.78	0.002
	Taking walks	2.60	2.01	3.37	< 0.001
	Biking	1.16	0.84	1.61	0.365
	Hiking	1.18	0.93	1.49	0.168
	Running/Nordic Walking	0.88	0.68	1.15	0.363
	Other outdoor sport	1.09	0.82	1.45	0.565
	Forestry/logging	1.31	0.96	1.78	0.085
	Bird watching	0.76	0.53	1.09	0.139
	Fishing	0.92	0.48	1.80	0.818
	Hunting	2.93	1.24	6.90	0.014
	Bee keeping	1.10	0.48	2.54	0.815
Tick protective strategies***	Using 0-1 strategies	1.00			
	Using 2-4 strategies	0.54	0.42	0.70	< 0.001
	Using 5-7 strategies	0.35	0.24	0.53	< 0.001
Garden properties	Skin contact with greenery	1.52	1.00	2.32	0.050
	Sighting animals	0.97	0.73	1.29	0.826
	Tick control	1.05	0.46	2.43	0.900
	Keeping lawns mowed	0.62	0.43	0.90	0.012
	Regular removal of fallen leaves	0.93	0.72	1.20	0.559
Garden's proximity to forest	> 1km	1.00			
	1000–751m	1.19	0.77	1.86	0.434
	750–501m	0.82	0.47	1.43	0.482
	500–250m	1.60	1.15	2.23	0.005
	< 250m	2.59	1.86	3.60	< 0.001
**Parameters only used for adjustment**					
	age (1-year steps)	0.99	0.99	1.00	0.012
	sex	0.89	0.71	1.12	0.325
	partial TBE vaccination (≥ 1 dose)	0.14	0.10	0.19	< 0.001
General tick bites	never	1.00			
	last bite > 1 year ago	0.15	0.10	0.22	< 0.001
	1-2 bites in last year	0.10	0.07	0.14	< 0.001
	≥ 3 bites in last year	0.52	0.36	0.76	0.001
Matching regions	Karlsruhe1	0.69	0.26	1.86	0.464
	Freiburg	0.86	0.38	1.96	0.723
	Tübingen1	1.05	0.42	2.66	0.912
	Karlsruhe2	0.85	0.35	2.05	0.722
	Tübingen2	0.91	0.40	2.05	0.811
	Stuttgart2	1.11	0.46	2.69	0.818
	Oberbayern1	0.75	0.29	1.89	0.536
	Niederbayern	0.92	0.35	2.37	0.858
	Oberpfalz	0.74	0.32	1.74	0.493
	Oberfranken	0.87	0.34	2.22	0.764
	Mittelfranken	0.75	0.31	1.80	0.520
	Unterfranken	0.70	0.20	2.50	0.583
	Schwaben1	1.17	0.20	6.89	0.865
	Oberbayern2	0.86	0.36	2.05	0.733
	Schwaben2	0.85	0.33	2.16	0.730
Season of exposure period (cases) or reference period (controls)	January–March	1.00			
	April–June	0.78	0.19	3.14	0.723
	July–September	0.74	0.18	2.99	0.674
	October–December	0.56	0.14	2.36	0.434

OR = odds ratio; CI = confidence interval.

*activities include (in descending frequency): biking, hiking, running/Nordic Walking, other outdoor sport, forest-/wood working, bird watching, fishing, hunting, bee keeping.

**Individual activities were dichotomised from original 5-point scale. A cut-off at 4×/week was applied for common activities, for which <50% of participants answered "never" (gardening, taking walks, biking). A cut-off at 1×/week was applied for rarer activities, for which >50% answered "never" (other 6 activities).

***regularly applying tick-protective strategies (e.g. tick repellents) during exposure time (cases) or reference time (controls).

**** English translations: Abitur = general qualification for university entrance; Fachabitur = subject-related entrance qualification; Realschulabschluss = intermediate school-leaving certificate; Hauptschulabschluss = completion of compulsory basic secondary schooling.

Table S3. Animal species sighted in gardens, as reported by TBE cases and controls.

**Animal species**	**Controls % sighted**	**Cases % sighted**	**p-value**
	n = 619	*n* = 346	Chi²
Hedgehogs	67.4%	70.5%	0.312
Squirrels	53.3%	52.6%	0.832
Martens	27.9%	27.7%	0.946
Deer/stags	24.1%	32.4%	0.010
Foxes	22.9%	27.7%	0.097
Wild boars	15.2%	18.8%	0.148
Hares/rabbits	13.2%	15.0%	0.443
Moles	6.5%	12.7%	0.001
Other forest animals	10.7%	5.2%	0.004
Mice	1.3%	7.5%	< 0.001

Table S4. Proportion of TBE cases (*n* = 558) reporting selected factors associated with TBE in 2020 compared to 2018/2019.

**Covariate**		**Cases notified 2018-19**		**Cases notified 2020**		**p-value**
		n	*n*	*n*	%	
General exposures	Rural residence	159	47.0%	109	49.5%	0.579
	Dog ownership	96	28.4%	71	32.3%	0.329
	Not staying on paths	85	25.1%	48	21.8%	0.316
	Raw milk intake	68	20.1%	37	16.8%	0.547
Gardening	< 1× / week (reference)	85	25.1%	39	17.7%	
	1-3× / week	97	28.7%	70	31.8%	0.128
	≥ 4× / week	156	46.2%	110	50.0%	
Taking walks	<1× / week (reference)	105	31.1%	25	11.4%	
	1-3× / week	121	35.8%	104	47.3%	< 0.001
	≥ 4× / week	112	33.1%	91	41.4%	
Other outdoor activities*	< 1× / week (reference)	93	27.5%	43	19.5%	
	1-3× / week	140	41.4%	103	46.8%	0.098
	≥ 4× / week	105	31.1%	74	33.6%	
Tick protection	Using 0-1 strategies**	113	33.4%	80	36.4%	
	Using 2-4 strategies**	192	56.8%	124	56.4%	0.528
	Using 5-7 strategies**	33	9.8%	16	7.3%	

*activities include (in descending frequency): biking, hiking, running/Nordic Walking, other outdoor sport, forest-/wood working, bird watching, fishing, hunting, bee keeping.

**regularly applying tick-protective strategies (e.g. tick repellents) during exposure time (cases) or reference time (controls).
